# Immune microenvironment analysis and novel biomarkers of early-stage lung adenocarcinoma evolution

**DOI:** 10.3389/fonc.2023.1150098

**Published:** 2023-06-23

**Authors:** Jun Liu, Yaxin Ji, Xiaodan Weng, Wei Shao, Jiaping Zhao, Hanlin Chen, Lu Shen, Fufeng Wang, Qi Meng, Xue Wu, Xiaonan Wang, Qiuxiang Ou, Honggang Ke

**Affiliations:** ^1^ Department of Chemotherapy, Affiliated Hospital of Nantong University, Nantong, China; ^2^ Department of Thoracic Surgery, Affiliated Hospital of Nantong University, Medical School of Nantong University, Nantong, China; ^3^ Department of Thoracic Surgery, Affiliated Hospital of Nantong University, Nantong, China; ^4^ Geneseeq Research Institute, Nanjing Geneseeq Technology Inc., Nanjing, China

**Keywords:** early-stage lung adenocarcinoma, RNA-sequencing, immune microenvironment, tumor evolution, biomarkers

## Abstract

**Background:**

Lung cancer is the deadliest and most diagnosed type of cancer worldwide. The 5-year survival rate of lung adenocarcinoma (LUAD) dropped significantly when tumor stages advanced. Patients who received surgically resecting at the pre-invasive stage had a 5-year survival rate of nearly 100%. However, the study on the differences in gene expression profiles and immune microenvironment among pre-invasive LUAD patients is still lacking.

**Methods:**

In this study, the gene expression profiles of three pre-invasive LUAD stages were compared using the RNA-sequencing data of 10 adenocarcinoma in situ (AIS) samples, 12 minimally invasive adenocarcinoma (MIA) samples, and 10 invasive adenocarcinoma (IAC) samples.

**Results:**

The high expression levels of PTGFRN (Hazard Ratio [HR] = 1.45; 95% Confidence Interval [CI]: 1.08-1.94; log-rank P = 0.013) and SPP1 (HR = 1.44; 95% CI: 1.07-1.93; log-rank P = 0.015) were identified to be associated with LUAD prognosis. Moreover, the early LUAD invasion was accompanied by the enhancement of antigen presentation ability, reflected by the increase of myeloid dendritic cells infiltration rate (Cuzick test P < 0.01) and the upregulation of seven important genes participating in the antigen presentation, including HLA-A (Cuzick test P = 0.03), MICA (Cuzick test P = 0.01), MICB (Cuzick test P = 0.01), HLA-DPA1 (Cuzick test P = 0.04), HLA-DQA2 (Cuzick test P < 0.01), HLA-DQB1 (Cuzick test P = 0.03), and HLA-DQB2 (Cuzick test P < 0.01). However, the tumor-killing ability of the immune system was inhibited during this process, as there were no rising cytotoxic T cell activity (Cuzick test P = 0.20) and no increasing expression in genes encoding cytotoxic proteins.

**Conclusion:**

In all, our research elucidated the changes in the immune microenvironment during early-stage LUAD evolution and may provide a theoretical basis for developing novel early-stage lung cancer therapeutic targets.

## Introduction

Lung cancer is the most deadly and commonly diagnosed type of cancer worldwide (1). Among all lung cancer patients, lung adenocarcinoma (LUAD) is the most prevalent histological type with a 5-year survival rate of only 15% (2). However, if LUAD can be detected in the pre-invasive stages, the survival rate after surgery will increase significantly. Based on the classification standard suggested by the International Association for the Study of Lung Cancer (IASLC), the adenocarcinoma *in situ* (AIS) is defined as a localized adenocarcinoma invasion with a diameter of less than 3 cm, while the minimally invasive adenocarcinoma (MIA) is a small solitary adenocarcinoma (less than 3 cm in diameter) with less than 5 mm invasion (3).

AIS, MIA, and invasive adenocarcinoma (IAC) patients had different prognostic performances shown in previous studies. AIS and MIS patients can achieve a 5-year overall survival rate of nearly 100% after surgery, and the 5-year overall survival rate gradually decreases as cancer stages advance (4). It is reported that surgically resecting LUAD at the pre-invasive stages (AIS and MIA stages) can dramatically improve life expectancy with an approximate gain in life years of 10.8 years (5). As AIS and MIA patients displayed a similar prognosis pattern, when compared with the IAC group, most studies treated AIS and MIA as one pre-invasive group. However, in the fifth edition of the WHO classification guide, AIS is not considered an invasive-adenocarcinoma (ADC) condition (6). Hence, grouping AIS and MIA as one stage may no longer be appropriate when studying early LUAD evolution. Moreover, AIS and MIA patients exhibited various difference in the clinicopathological features and long-term prognosis. AIS patients had higher pure ground-glass nodules rates (7), better disease-specific 10-year survival rates (8), and less recurrence events (9).

Despite all the benefits of early diagnosis, the trivial morphological differences between AIS and MIA bring struggles in discriminating them clinically. The limited knowledge of the structural characteristics of lung cancer tissues and the absence of proven diagnostic biomarkers are the principal causes of false classifications. Now, the diagnosis of AIS or MIA requires a histologically complete sampling of the tumor (10). A non-invasive way of diagnosing AIS and MIA in potential early-invasive LUAD patients is urgently needed.

There are several studies that reported the genetic landscapes and immune microenvironment of pre-invasive LUAD. EGFR, BRAF, ERBB2, TP53, KRAS, etc., were recognized as significantly mutated genes in the AIS and MIA groups. Immune infiltration was identified to relate to arm-level copy number variations of 6p (11). Genes regulating cell mobility, gap junction, and metastasis were not driver mutations in pre-invasive LUAD. EGFR was the most common genetic alteration in AIS, MIA, and invasive adenocarcinoma (IAC) groups, while TP53 was only detected in MIA and IAC. IAC showed a higher CD8 infiltration (12). However, most previous studies are based on the DNA, protein, or the cellular level. The study focusing on the differences in gene expression profiles among AIS, MIA, and IAC patients is still lacking.

In this study, we comprehensively compared the gene expression profiles of AIS, MIA, and IAC patients using the RNA sequencing data of a total of 32 samples and illustrated the molecular mechanisms behind the evolution of early lung adenocarcinoma.

## Materials and methods

### Study cohort

A total of 31 patients with pulmonary nodules undergoing thoracic surgery in Nantong University Affiliated Hospital were prospectively recruited from July 2020 to March 2021. Patients were included according to the following criteria (1): over 18 years old (2); Preoperative CT image showed single ground-glass nodule, with a size of less than or equal to 30mm (3); Pathologically confirmed adenocarcinoma with no other concurrent malignancies. According to the pathological characteristics before and after infiltration, the enrolled cases were divided into three groups, namely AIS, MIA, and IAC. The postoperative pathological examination was performed by at least 2 experienced pathologists. The gene expression differences between the adjacent two groups, including AIS-MIA and MIA-IAC, were compared. This study was approved by the ethics committee of the Affiliated Hospital of Nantong University, registered number 2022-L144.

### Sample collection

From fresh lung specimens, the tumor tissues of about 3mm*3mm*3mm in size were obtained by trimming the core areas of the nodules. The tissues were immediately stored in a -80°C refrigerator. The sample was included in the study after the pathological results of the Hematoxylin-Eosin (HE) staining were confirmed to meet the standard. The centrifuge tubes containing the samples were buried in dry ice waiting for RNA sequencing.

### RNA extraction and sequencing library preparation

The RNA was extracted using RNeasy FFPE Kit (Qiagen lnc.). The RNA purity was checked by evaluating A260/280 and A260/230 ratios using Nanodrop 2000 (Thermo Fisher Scientific). The quantity of the extracted RNA was evaluated using a Qubit 3.0 fluorometer (Thermo Fisher Scientific). Sequencing libraries were prepared using the KAPA Stranded RNA-seq Kit with RiboErase (KAPA Biosystems) according to the manufacturer’s suggestions. Briefly, ribosome RNA (rRNA) and DNA were removed using RiboErase and DNase Digestion, respectively. dUTP strand-specific RNA-seq libraries were constructed and were preliminarily quantified using Qubit2.0. The library was diluted to 1 ng/μl and the insert size was examined using the Agilent 2100. The library was quantified by qPCR (effective concentration > 3nM). RNA-sequencing was performed on the Illumina HiSeq4000 platform using PE150 sequencing chemistry (Illumina) with an average throughput of 50M reads/sample.

### RNA sequencing

The raw image data was converted into FASTQ format using the software bcl2fastq2 (v2.19.). The N bases, low-quality data (score < 15), adapter reads, and reads pairing with rRNA or tRNA were removed using Trimmomatic (13). The percentage of clean reads of all 32 samples ranged from 98.2% to 99.3%, with a median number of clean reads of 31.4 million reads per sample (ranging from 23.4 to 41.0 million reads per sample) and a median Q30 rate of 93.0% (ranging from 92.3% to 93.5%). All clean reads were aligned to the reference human genome (hg19) using STAR V2.5.2b (14). The gene expressions were quantified and normalized by RSEM (15). In short, the expectation maximization algorithm was applied to optimally assign reads mapped to multiple transcripts and generate FPKM (Fragments Per Kilobase Million) data. Furthermore, the TPM (transcripts per million) values of all genes were introduced to make comparisons between samples. The differential expressed gene analysis was performed using DESeq2 (16) with the negative binomial distribution test. Genes considered statistically significant differentially expressed between MIA and IAC by DESeq2 (false discovery rate [FDR] < 0.1) with a fold change (FC) absolute value larger than 1.5 were deemed to be differentially expressed genes (17, 18). Only those differentially expressed genes between MIA and IAC that did not show opposite trend between AIS and MIA proceeded to further analysis (**Figure S1**).

### KEGG pathway analysis

For the screened differentially expressed genes, KEGG (19) enrichment analysis was performed using clusterProfiler v3.2.14 package in R (v3.5.3). The results were visualized using the ggplot2 package in R (v3.5.3).

### Immune microenvironment analysis

For immune-related analysis, the TPM expressions of a total of 74 immune regulation-related genes were analyzed, which can be classified as antigen presentation, cell adhesion, co-inhibitor, co-stimulator, ligand, receptor, and others (20). The results were visualized using the ComplexHeatmap package in R (v3.5.3). To evaluate the immune effector activity of our samples, the cytolytic activity score was calculated as the geometric mean of the TPM expression levels of GZMA and PRF1 genes (21). A higher cytolytic activity score was considered to associate with an immunosuppressive tumor microenvironment (22).

To further investigate the landscape of immune cell infiltration of our samples, we analyzed RNA expression data through the Quantiseq algorithm (23). The infiltration fraction of ten immune cell types were calculated, including B cells, M1 macrophages, M2 macrophages, monocytes, neutrophils, natural killer (NK) cells, CD4+T cells, CD8+ T cells, regulatory T cells, and myeloid dendritic cells (MDC). Among them, CD4+ T cells and monocytes were not included in the statistical analysis due to underrepresented results.

### Statistical analysis

The relative TPM expression of the MHC Class I, Class II, and cytotoxin-associated genes were calculated as the TPM expression of each gene divided by the average TPM values of all genes. The non-parametric Wilcoxon’s rank-sum test was used to assess the differences between two groups. Comparisons among three or more groups were performed using Kruskal–Wallis H test. Trend analysis between different groups was conducted using the Cuzick’s trend test in the “PMCMRplus” package in R. A P value less than 0.05 was considered to be statistically significant. All tests were done in R (v3.5.3).

### Prognosis and gene expression validation using TCGA cohorts

Results validation was conducted using the RNA sequencing data of 504 cases with overall survival data from The Cancer Genome Atlas Program (TCGA) lung adenocarcinoma cohort (https://tcga-data.nci.nih.gov/tcga). The median value of gene expression was used as the threshold to separate the high expression subgroup from the low expression subgroup, and the KM curve was plotted using the OS data. The p-value (log-rank test), the 95% confidence interval, and the hazard ratio (HR) were calculated. All statistical analysis and plotting were performed using R (v3.5.3).

## Results

### Study cohort and clinical characteristics

**Figure 1:**
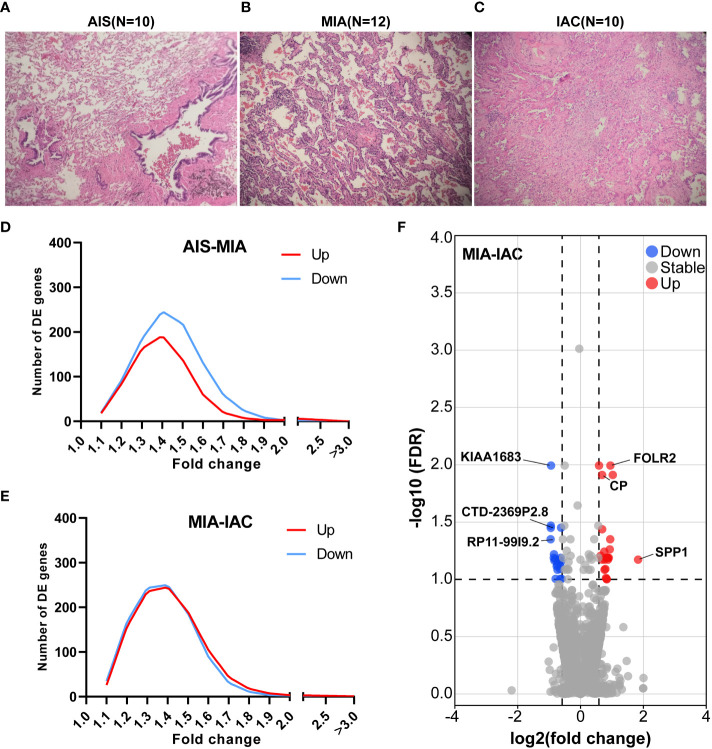
The relative TPM expressions of MHC Class I and Class II genes, and the cytolytic activity scores in the AIS, MIA, and IAC. **(A)** The relative TPM expressions of MHC Class I genes in the AIS, MIA, and IAC. **(B)** The relative TPM expressions of MHC Class II genes in the AIS, MIA, and IAC. **(C)** The bar plot showing the mean cytolytic activity scores of AIS, MIA, and IAC patients with error bars. **(D)** The bar plot showing the cytolytic activity scores of each AIS, MIA, and IAC patient. Note: *: P < 0.05; **: P < 0.01 (Comparisons between two groups were performed using the Wilcoxon test, trends were tested using the Cuzick trend test).

The RNA-sequencing data of 32 samples collected from 31 patients were analyzed, of which 10 were adenocarcinoma *in situ* (AIS) samples from 9 patients, 12 minimally invasive adenocarcinoma (MIA) samples, and 10 invasive adenocarcinoma (IAC) samples (**Figures 1**). The enrolled IAC tumor samples were in the early stages with relatively small lesions, which, judging from the sizes, may have just been transformed from MIAs. Among all these samples, 65.6% of them were from female patients, and there was no significant discrepancy in age and sex distribution among the three groups (**Table 1**, **Table S1**).

**Table 1:** Clinicopathologic features of the enrolled patients and samples.

Characteristics	AIS (n = 10)	MIA (n = 12)	IAC (n = 10)	P value
Sex				0.34
Female	8 (80%)	6 (50%)	7 (70%)	
Male	2 (20%)	6 (50%)	3 (30%)	
Age(Median)				0.46
> 57	3 (30%)	7 (58.3%)	5 (50%)	
≤ 57	7 (70%)	5 (41.7%)	5 (50%)	
Tumor diameter(mm)				
Median (range)	9.5 (8~15)	11.5 (6~15)	15 (8~21)	
T stage				
Tis	10 (100%)	0	0	
T1 mi	0	12 (100%)	0	
T1 a-c	0	0	9 (90%)	
T2	0	0	1 (10%)	

### Differential expressed genes and pathway analysis

Seventy-two genes were identified to be up-regulated in MIA samples compared to AIS samples while 192 genes were down-regulated. Meanwhile, 150 genes were identified up-regulated in IAC samples compared to MIA samples and 102 genes were down-regulated (P < 0.05, **Figure 1**). After multiple comparison corrections (FDR) and fold change filtering, only genes differentially expressed between MIA and IAC groups (FDR < 0.1 & |FC| > 1.5) while showing no significant opposite trend between AIS and MIA groups were kept for further analysis (**Figure S1**). Hence, there were a total of 23 up-regulated genes and 21 down-regulated genes throughout the early LUAD (AIS to MIA to IAC) evolution process (**Table S2**). Interestingly, in the pathway enrichment analysis, the down-regulated genes were enriched in interleukin-17 (IL-17) signaling pathway (FDR = 0.003, **Figure S3**).

**Figure 2:**
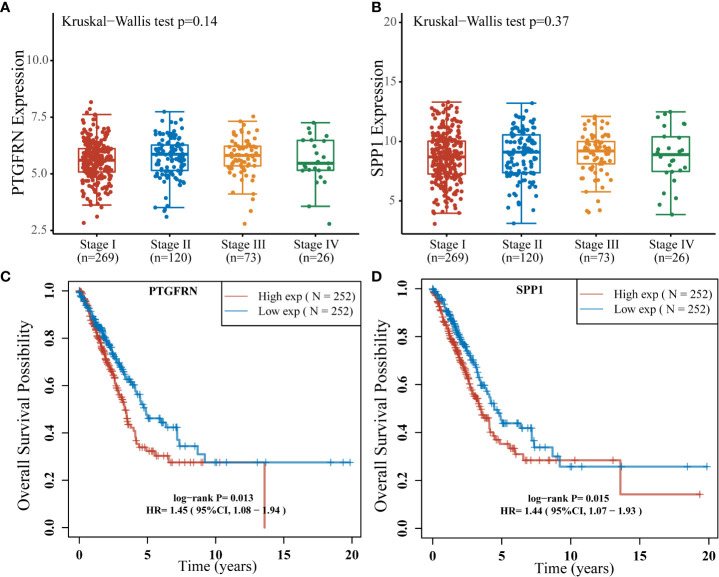
The expression levels of 9 major cytotoxic protein encoding genes and the infiltration rates of multiple immune cells in the AIS, MIA, and IAC patients. **(A)** The expression levels of 9 major cytotoxic protein encoding genes cells in the AIS, MIA, and IAC patients. The infiltration rates of immunes cells in the AIS, MIA, and IAC patients, including **(B)** myeloid dendritic cells (MDC); **(C)** M1 macrophages; **(D)** M2 macrophages; **(E)** neutrophils; **(F)** natural killer (NK) cells; **(G)** regulatory T cells (T-reg); **(H)** CD8+ T cells; **(I)** B cells. Note: *: P < 0.05; **: P < 0.01 (Comparisons between two groups were performed using the Wilcoxon test, trends were tested using the Cuzick trend test).

To uncover potential prognostic markers which play vital roles in early LUAD development from MIA to IAC, we utilized the TCGA LUAD data (see Methods) to examine those 50 genes with the highest expression fold changes during the transition. The cohort was separated into high-expression and low-expression subgroups based on the median expression levels of these genes. Among them, the high expression levels of PTGFRN (hazard ratio [HR] = 1.45; 95% confidence interval [CI]: 1.08-1.94; log-rank P = 0.013) and SPP1 (HR = 1.44; 95% CI: 1.07-1.93; log-rank P = 0.015) were found to be significantly associated with worse prognosis in the TCGA LUAD cohort (**Figure 2**). Moreover, there was no significant correlation between the expression of SPP1 or PTGFRN and the tumor stages after infiltration (**Figure 2**).

### Immune microenvironment analysis

**Figure 3:**
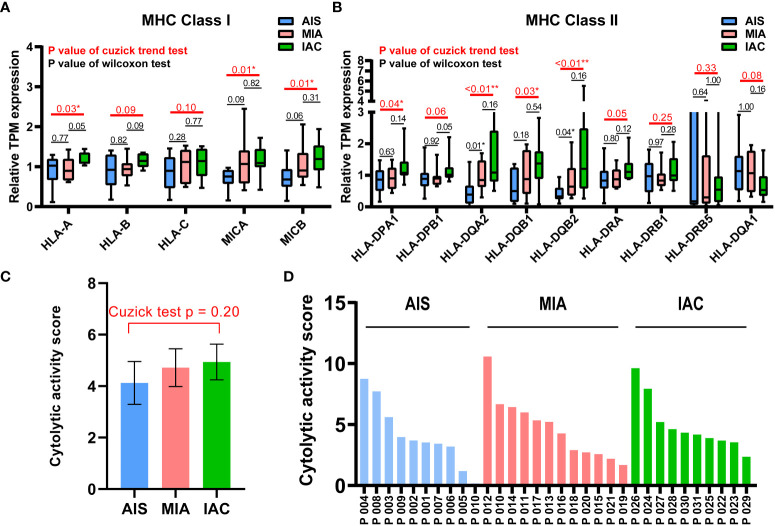
Differentially expressed gene analysis between adenocarcinoma in situ, minimally invasive adenocarcinoma, and invasive adenocarcinoma. Microphotographs showing the histological morphology of **(A)** adenocarcinoma *in situ* (AIS), N=10; **(B)** minimally invasive adenocarcinoma (MIA), N=12; and **(C)** invasive adenocarcinoma (IAC), N=10. **(D)** The fold change distribution of upregulated and downregulated genes between AIS and MIA patients. **(E)** The fold change distribution of upregulated and downregulated genes between MIA and IAC patients. **(F)** The volcano plot showing differential expressed genes between MIA and IAC patients (absolute value of fold change > 1.5, FDR < 0.1).

The expression of 74 Immunomodulating-related genes, which can be classified into 7 categories, including antigen presentation, cell adhesion, co-inhibitor, co-stimulator, ligand, receptor, and other (20), were analyzed. As shown in **Figure S2**, the antigen presentation-related genes showed the most pronounced and consistent regulation trend throughout the early LUAD evolution process. Major histocompatibility complex (MHC) class I and MHC class II genes are major types of antigen presentation genes. Among MHC class I genes, HLA-A (Cuzick test P = 0.03), MICA (Cuzick test P = 0.01), and MICB (Cuzick test P = 0.01) showed a significant increasing trend in expression levels from AIS to MIA to IAC, while some MHC Class II genes, including HLA-DPA1 (Cuzick test P = 0.04), HLA-DQA2 (Cuzick test P < 0.01), HLA-DQB1 (Cuzick test P = 0.03), HLA-DQB2 (Cuzick test P < 0.01), also manifested the same trend (**Figures 3**). The rest of the MHC class I and class II genes displayed a similar tendency however not reaching the statistical significance threshold.

**Figure 4:**
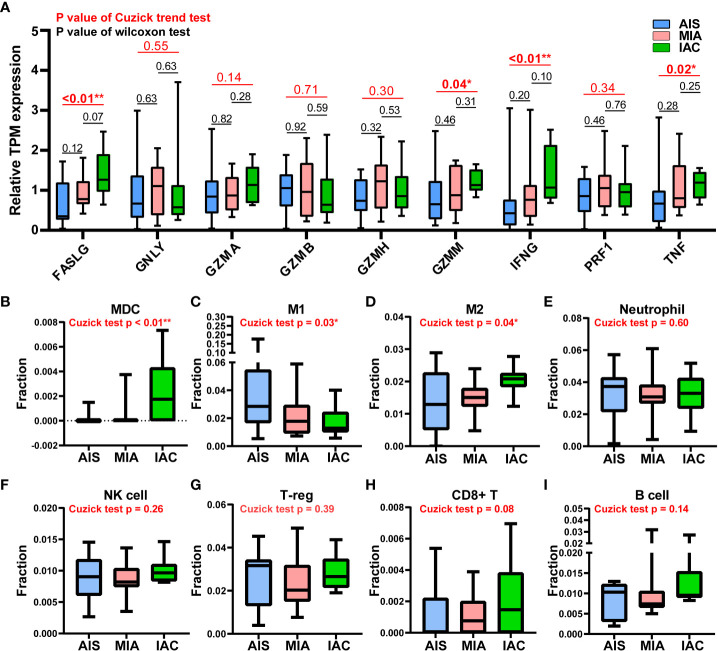
Stages and survival analysis about the SPP1 and PTGFRN expression levels in lung adenocarcinoma patients using the TCGA LUAD cohort. **(A)** PTGFRN expression levels in lung adenocarcinoma patients of different stages (Kruskal–Wallis P = 0.14). **(B)** SPP1 expression levels in lung adenocarcinoma patients of different stages (Kruskal–Wallis P = 0.37). **(C)** Kaplan-Meier overall survival curves of lung adenocarcinoma patients according to different PTGFRN expression levels (Hazard Ratio [HR] = 1.45; 95% Confidence Interval [CI]: 1.08-1.94; log-rank P = 0.013). **(D)** Kaplan-Meier overall survival curves of lung adenocarcinoma patients according to different SPP1 expression levels (HR = 1.44; 95% CI: 1.07-1.93; log-rank P = 0.015).

Cytolytic activity score was defined as the geometric mean of GZMA and PRF1 expression, which is a new index of cancer immunity representing the CD8+ T cell activation level (21). No statistically significant trend was observed in cytolytic activity scores from AIS to MIA to IAC (Cuzick test P = 0.20, **Figure 3**). As the immune cells kill tumor cells by releasing small cytotoxic proteins that cause apoptosis in target cells (24), we analyzed the expression profiles of 9 major genes encoding cytotoxic proteins, including FASLG, GNLY, GZMA, GZMB, GZMH, GZMM, IFNG, PRF1, TNF (25). FASLG, GZMM, IFNG, and TNF were significantly upregulated throughout the transition process from AIS to MIA to IAC (P < 0.01, P = 0.04, P < 0.01, P = 0.02, respectively; **Figure 4**).

Based on a deconvolution algorithm, Quantiseq uses mRNA expression data to predict the composition of different types of immune cells in tumor samples. We found that when the tumor transformed from MIA to IAC, the myeloid dendritic cells (MDC) infiltration rate was significantly increased (P < 0.01). M1 and M2 macrophages displayed the opposite trend of infiltration rate, as M1 macrophages infiltration rate increased (P = 0.03) and M2 macrophages decreased (P = 0.04) from AIS to MIA to IAC. For other immune cell types, there was no significant difference in infiltration rates during the transition (**Figures 4**).

## Discussion

Based on the RNA-sequencing data of 32 samples, we comprehensively compared the gene expression profiles among AIS, MIA, and IAC patients. The IL-17 signaling pathway involved in producing T-cells (26), were found to be down-regulated throughout early lung adenocarcinoma evolution process, which may indicate a reduced tumor-killing ability during the process. In our study, two genes of which the expression was up-regulated in the MIA-IAC conversion, SPP1 and PTGFRN, were associated with the prognosis in the TCGA LUAD cohort (log-rank P = 0.015 and 0.013, respectively). The elevated expression of SPP1 and PTGFRN is an event that occurs when LUADs transit from pre-infiltration to post-infiltration and is a stage-independent prognostic factor. SPP1 had a high expression profile in the TCGA lung cancer cohort compared to healthy subjects and was associated with poor prognosis in lung cancer patients. It was validated at the cellular level that SPP1 facilitates lung cancer progression, migration, and invasion (27). PTGFRN was also shown to be overexpressed in tumors and was recognized as an essential part of angiogenesis, which is a necessary process in tumor proliferation (28). The inhibition of PTGFRN lifted the radiosensitivity of glioblastoma tumors and declined tumor growth (28). Our results suggest that the high expression levels of SPP1 and PTGFRN may be a key component in the LUAD early invasions and can lead to a more malignant tumor condition.

Among the MHC class I and class II genes, seven important genes participating in the antigen presentation were found to be significantly upregulated during the AIS-MIA-IAC conversion, including HLA-A (Cuzick test P = 0.03), MICA (Cuzick test P = 0.01), MICB (Cuzick test P = 0.01), HLA-DPA1 (Cuzick test P = 0.04), HLA-DQA2 (Cuzick test P < 0.01), HLA-DQB1 (Cuzick test P = 0.03), and HLA-DQB2 (Cuzick test P < 0.01),. An elevated expression can be witnessed for the rest of the MHC class I and class II genes from AIS to MIA to IAC, though not reach the statistically significant threshold. These findings may indicate a rising antigen-presenting ability in the LUAD early invasion process. Moreover, the infiltrating rate of myeloid dendritic cells (Cuzick test P < 0.01), which are responsible for capturing, processing, and presenting neoantigens to T cells (29), increased during the early LUAD evolution. However, there was no increasing trend in cytolytic activity score (Cuzick test P = 0.20), representing no rising cytotoxic T cell activity from AIS to IAC. Meanwhile, there was no sign of increasing expression in cytotoxic proteins. There was no elevated CD8+ T cell infiltrating activity detected as well. In line with our results, it has been proven that mutations of genes associated with the cell mobility, gap junction, and metastasis were enriched in MIA and IAC phases instead of in AIS, accompanied by an increase of TMB (12). Hence, during the early invasion, the number of neoantigens escalated, leading to an upregulated antigen-presenting ability. Meanwhile, during the transformation from pre-invasive to post-invasive lung cancer, a significant decrease in the anti-cancer lymphocytes, specially CD8+T, was observed (30). Moreover, the gene expression of cell destruction proteins, including ENTPD1, GZMB, and PRF1, was inhibited (31). Therefore, we think the early invasion process of lung adenocarcinoma is accompanied by the enhancement of antigen presentation ability, but the tumor-killing ability of the immune system is inhibited, which leads to the occurrence of lung adenocarcinoma invasion.

We also observed that M1 macrophages and M2 macrophages showed a continuous decreasing and increasing trend during the development of early lung adenocarcinoma (Cuzick test P = 0.03 and 0.04, respectively). M1 macrophages can kill tumor cells by mediating antibody-dependent cytotoxicity or by mediating cytotoxicity directly. However, M2 macrophages are considered to participate in stimulating tumor proliferation and invasion (32). It is suggested that the invasion of lung adenocarcinoma will be accompanied by the weakening of the anti-tumor process mediated by macrophages.

Previous studies reported that neoadjuvant immunochemotherapy could lead to substantial rates of major pathologic response and pathologic complete response (33). The MPR and pathological complete response rates were significantly higher for early-stage lung cancer patients who received neoadjuvant immunotherapy and chemotherapy than who only received chemotherapy (34). Our findings also supported the administration of immunotherapy in early-stage LUAD, as the tumor-killing ability of the immune system is inhibited while LUAD stages advance, which may undermine the efficacy of immunotherapy.

However, worth pointing out that, it is of great importance to map these markers and identify marker expressing cells in different stages including AIS, MIA and IAC to understand immune microenvironment and the impact of those genes in the LUAD progression. PTGFRN was previously shown to have membranous and cytoplasmic expressions in most tissues at both transcriptional and translational levels (www.proteinatlas.org). More importantly, PTGFRN expression was demonstrated to be associated with the metastatic status of lung cancer (35). A previous study by Karhemo et al., reported that PTGFRN showed significantly higher expression in the metastatic cancer cells than in non-metastatic cancer cells, and they verified the result in cell lines and *in vivo* using proteomics analysis (LC-MS/MS) and IHC staining, respectively (36). According to the Human Protein Atlas (www.proteinatlas.org), the SPP1 showed cytoplasmic expression only in a subset of tissues, most enriched in renal tubules and gallbladders. However, the overexpression of SPP1 was identified in various tumors, including breast, bladder, colorectal, head, neck, liver, lung, and esophageal cancers (37), and was considered a negative prognostic biomarker (38). Regarding the MHC-Class I (MHC-I) and MHC-Class II (MHC-II) molecules, MHC-I molecules are produced by the majority of nucleated cells and are responsible for presenting peptide antigens derived from within the cell to CD8+ T cells. Conversely, MHC-II molecules are mainly found in professional antigen-presenting cells (pAPC) such as dendritic cells (DC), B cells, and macrophages, and are responsible for presenting peptide antigens derived from outside the cell to CD4+ T cells (39). Tumor cells widely express MHC-I, while only a subset of tumors is capable of expressing MHC-II (39). In tumor cells, down-regulation on the cell surface of MHC-I can be observed to allow evasion from T-cell-mediated anti-tumor immunity, while sometimes tumors may temporarily up-regulate MHC-I expression to avoid the recognition of NK cells (40). Moreover, previous studies have shown that MICA and MICB, members of the MHC-I family, are frequently highly expressed in tumor cells and can be proteolytically shedded by tumors as an important immune evasion mechanism avoiding the recognition of NK cells (41, 42). MHC-II was found expressed in a diverse of human cancer, such as non–small cell lung cancer, colorectal cancer, breast cancer, ovarian cancer, and prostate cancer, in which tissue of origin showed no evidence of expressing MHC-II molecules (39). Tumor-specific MHC-II expression may result in recognition of cancers by the immune system and therefore is considered a positive indicator of immunotherapy efficacy and prognosis (39). Hence, future multiplex analyses, such as 10x Spacial transcriptomics and multiplex IF analysis, are needed to validate these biomarkers.

In all, this study comprehensively compared the gene expression profiles among AIS, MIA, and IAC patients using the RNA sequencing data. High expression levels of SPP1 and PTGFRN were identified to be associated with LUAD prognosis. Our data suggest that the early lung adenocarcinoma invasion is accompanied by the enhancement of antigen presentation ability, but the tumor-killing ability of the immune system is inhibited, which leads to the occurrence of lung adenocarcinoma invasion.

## Data availability statement

The data supporting this study's findings are deposited in the Genome Sequence Archive for Human (GSA-Human) repository (URL: https://ngdc.cncb.ac.cn/gsa-human/), accession number HRA003329.

## Ethics statement

The studies involving human participants were reviewed and approved by the ethics committee of the Affiliated Hospital of Nantong University, registered number 2022-L144. The patients/participants provided their written informed consent to participate in this study.

## Author contributions

Conceptualization: JL. Methodology: HK. Validation: XWE, WS, JZ, YJ. Formal analysis: JL, HC, QM, YJ. Data curation: LS, FW, XWU, XWA, QO. Supervision: HK. Original draft preparation: JL, YJ, XWE, WS, JZ, HC, QM, LS, FW, XWU, XWA, QO. Review and editing: HK, JL, HC, QO. All authors contributed to the article and approved the submitted version.
