# Supplementary material for: Immune microenvironment analysis and novel biomarkers of early-stage lung adenocarcinoma evolution
Source: Front Oncol. 2023 Jun 23;13:1150098. doi: 10.3389/fonc.2023.1150098 (PMC10328385; doi:10.3389/fonc.2023.1150098)
Supplement: Supplementary file 1 [file DataSheet_1.docx]

**
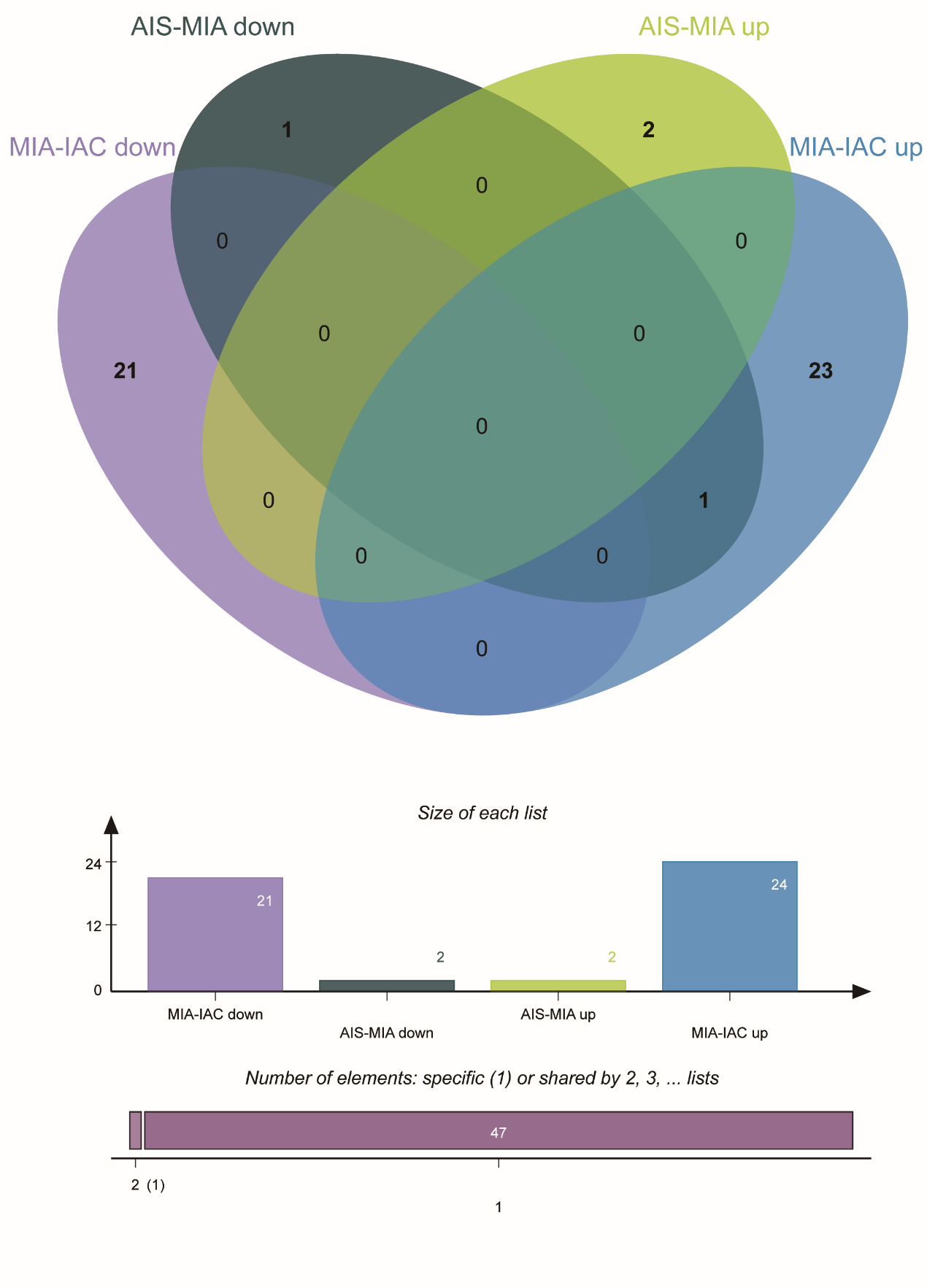
Figure S1: Distribution of differentially expressed genes among AIS, MIA, and IAC.** Only genes included in the red circles were included in the analysis (MIA-IAC up-regulated only, MIA-IAC up-regulated & AIS-MIA up-regulated, MIA-IAC down-regulated only, MIA-IAC down-regulated & AIS-MIA down-regulated)

**Figure S2: Heatmaps of immune regulation-related gene expressions.** (A) Heatmap of the expressions of immune regulation-related genes of all individuals; (B) Heatmap of the median expressions of immune regulation-related genes of each group.

**
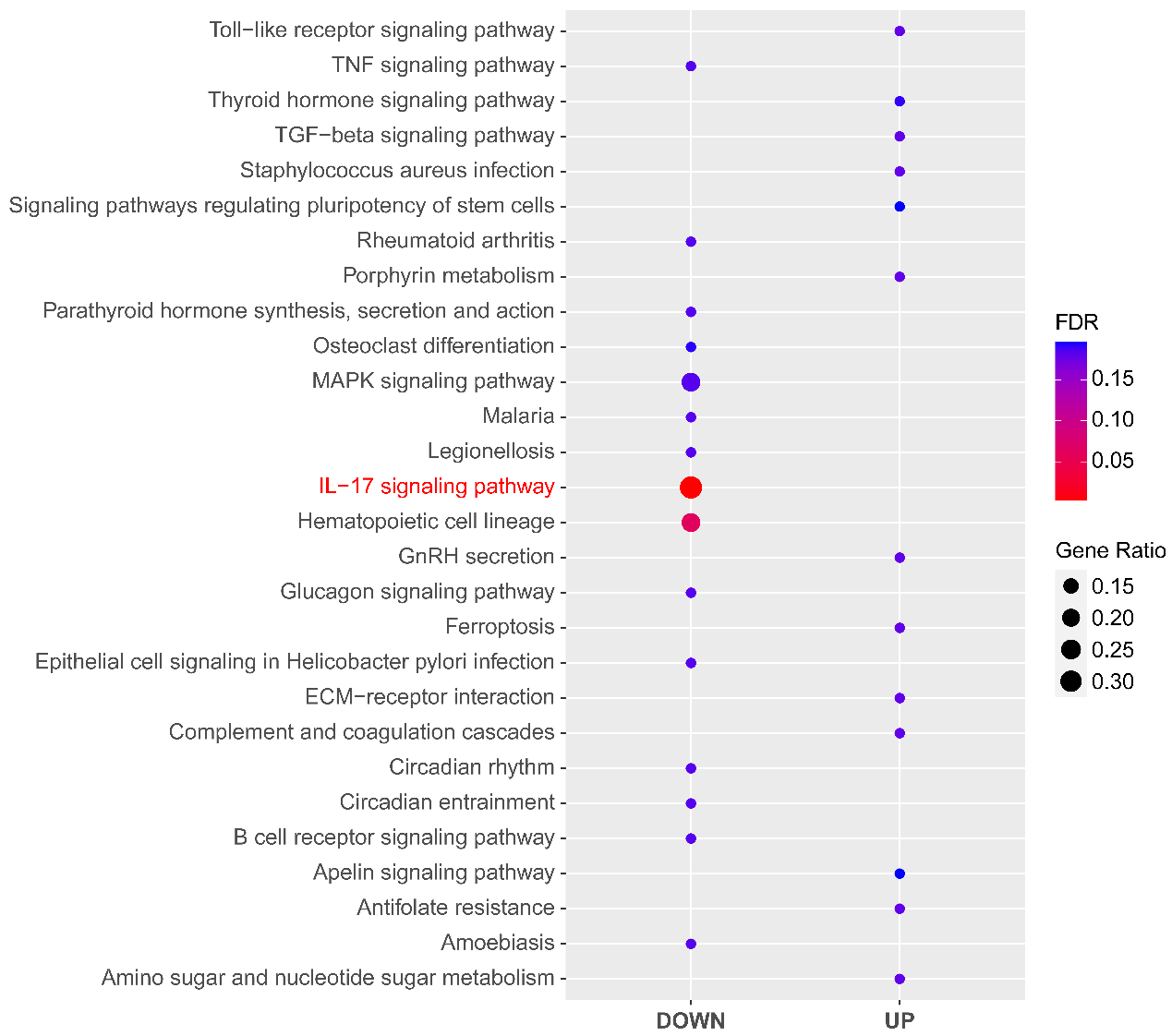
Figure S3: Up-regulated and down-regulated signaling pathways during early lung adenocarcinoma evolution.**


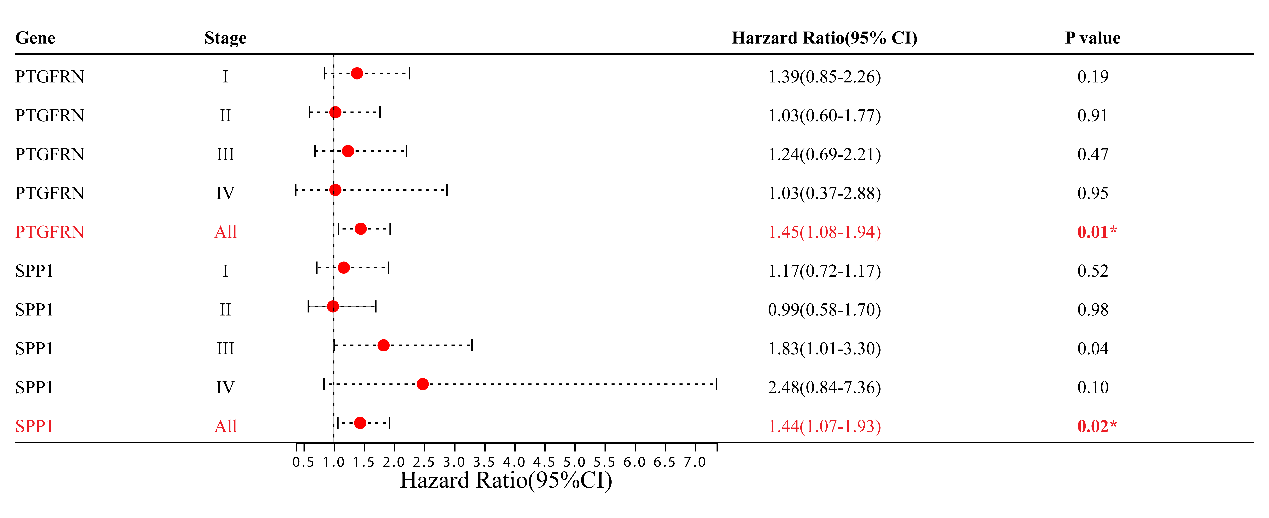


**Figure S4: Forest plot of lung adenocarcinoma patients according to PTGFRN and SPP1 expression levels in different tumor stages.**

**Table S1:**

**Clinicopathological characteristics of patients included in study (N = 31)**

| **Patient ID** | **Sex** | **Age** | **Tumor Diameter (mm)** | **Developmental stage** | **T** |
| --- | --- | --- | --- | --- | --- |
| P 001 | Female | 63 | 13 | AIS | Tis |
| P 002 | Female | 68 | 10 | AIS | Tis |
| P 003 | Male | 56 | 11 | AIS | Tis |
| P 004 | Female | 56 | 9 | AIS | Tis |
| P 005 | Female | 39 | 8 | AIS | Tis |
| P 006 | Female | 57 | 8 | AIS | Tis |
| P 007 | Female | 43 | 8 | AIS | Tis |
| P 008 | Female | 45 | 15 | AIS | Tis |
| P 009 | Male | 43 | 11 | AIS | Tis |
| P 010 | Female | 60 | 8 | AIS | Tis |
| P 010 | Female | 60 | 12 | MIA | T1 mi |
| P 011 | Male | 63 | 13 | MIA | T1 mi |
| P 012 | Female | 64 | 10 | MIA | T1 mi |
| P 013 | Female | 54 | 14 | MIA | T1 mi |
| P 014 | Male | 55 | 15 | MIA | T1 mi |
| P 015 | Female | 65 | 6 | MIA | T1 mi |
| P 016 | Female | 61 | 15 | MIA | T1 mi |
| P 017 | Male | 39 | 13 | MIA | T1 mi |
| P 018 | Female | 51 | 7 | MIA | T1 mi |
| P 019 | Male | 68 | 10 | MIA | T1 mi |
| P 020 | Male | 61 | 8 | MIA | T1 mi |
| P 021 | Male | 57 | 11 | MIA | T1 mi |
| P 022 | Male | 69 | 15 | IAC | T1b |
| P 023 | Female | 43 | 12 | IAC | T1b |
| P 024 | Female | 47 | 10 | IAC | T1a |
| P 025 | Male | 76 | 15 | IAC | T1b |
| P 026 | Female | 58 | 8 | IAC | T1a |
| P 027 | Female | 57 | 16 | IAC | T1b |
| P 028 | Female | 70 | 20 | IAC | T1b |
| P 029 | Female | 71 | 15 | IAC | T2 |
| P 030 | Male | 42 | 21 | IAC | T1c |
| P 031 | Female | 57 | 20 | IAC | T1b |

**Table S2:**

**Number of statistically significant differential expressed genes among each group**

|  | **MIA-IAC** | | **AIS-MIA** | |
| --- | --- | --- | --- | --- |
|  | **Up** | **Down** | **Up** | **Down** |
| **>3.0** | 1 | 0 | 0 | 0 |
| **2.0-3.0** | 1 | 0 | 0 | 1 |
| **1.9-2.0** | 3 | 4 | 1 | 0 |
| **1.8-1.9** | 5 | 1 | 1 | 0 |
| **1.7-1.8** | 6 | 4 | 0 | 0 |
| **1.6-1.7** | 6 | 4 | 0 | 1 |
| **1.5-1.6** | 2 | 8 | 0 | 0 |
| **1.4-1.5** | 2 | 5 | 0 | 0 |
| **1.3-1.4** | 1 | 6 | 0 | 0 |
| **1.2-1.3** | 4 | 2 | 0 | 0 |
| **1.1-1.2** | 3 | 0 | 0 | 0 |
| **1.0-1.1** | 1 | 3 | 0 | 0 |
| **Total** | 35 | 37 | 2 | 2 |
